# Supplementary material for: Augmenting a prognostic deep learning system for referable diabetic retinopathy and maculopathy with synthetic retinal images
Source: Commun Med (Lond). 2025 Dec 20;6:57. doi: 10.1038/s43856-025-01316-5 (PMC12830830; doi:10.1038/s43856-025-01316-5)
Supplement: Supplementary file 2 — Supplementary Information [file 43856_2025_1316_MOESM2_ESM.pdf]

Supplementary Material

Augmenting a prognostic deep learning system for referable diabetic retinopathy and maculopathy with synthetic retinal images

Paul Nderitu, FRCOphth[1,2*]; Joan M. Nunez do Rio, PhD[1]; Laura Webster[3]; Samantha S. Mann, FRCOphth[3,4]; David Hopkins, PhD[5,6]; Christos Bergeles, PhD[7]; Timothy L. Jackson, PhD, FRCOphth[1,2].

[1]Section of Ophthalmology, Faculty of Life Science and Medicine, King's College London, London, UK.

[2]King's Ophthalmology Research Unit, King's College Hospital, London, UK.

[3]South East London Diabetic Eye Screening Programme, Guy's and St Thomas' Foundation Trust, London, UK.

[4]Department of Ophthalmology, Guy's and St Thomas' Foundation Trust, London, UK.

[5]Department of Diabetes, School of Life Course Sciences, King's College London, London, UK.

[6]Institute of Diabetes, Endocrinology and Obesity, King's Health Partners, London, UK.

[7]School of Biomedical Engineering & Imaging Sciences, King's College London, London, UK.

TABLE OF CONTENTS

Supplementary methods 3
Study population and datasets 3
CCDM development hyperparameters 3
Prognostic DLS development and hyperparameters 4
Supplementary Table 1. Diabetic retinopathy grading definitions 5
Supplementary Table 2. Diabetic eye screening procedures 6
Supplementary Figure 1. Uncurated negative and positive synthetic retinal image case examples 7
Supplementary Table 3. Prognostic DLS AUPRC 8
References 9

SUPPLEMENTARY METHODS

Study population and datasets

Diabetic eye screening programme (DESP) data from south-east London DESP (SEL-DESP) were used for the development and internal test sets, whilst data from Birmingham DESP (B-DESP), a geographically independently screening service, were used for the external test set. The UK DESP follows established national screening committee grading definitions[1] and protocols[2] which are summarised in appendix table S1 and S2. All UK graders must undertake comprehensive training and are required to complete ~10 tests per year and need to attain an average sensitivity of >85% for referable DR detection on a varying test set.[3] Images from SEL-DESP and B-DESP were captured using a wide range of approved cameras (appendix table S2). Diabetic retinopathy (DR) and maculopathy grades, as documented by DESP graders, were extracted and used to derive the prognostic deep learning system (pDLS) training and evaluation labels. The UK DESP uses multi-level DR grading whereby a primary grader identifies the presence or absence of DR, with the exceptions of ungradable and proliferative DR cases which can be referred to the hospital eye service directly by the primary grader.[4] A secondary grader determines if DR is referable or non-referable in positive cases, and also reviews a random 10% sample of negative cases from primary grading as a quality assurance step to detect and quantify the rate of missed cases. A tertiary senior grader performs arbitration in cases of disagreement.

CCDM development hyperparameters

The AdamW optimizer minimized the mean squared error (MSE) between predicted and true noise added during the forward diffusion process, enabling the trained CCDM to generate synthetic images from random noise.[5] CCDM training hyperparameters included a constant learning rate of 0.0001, a batch size of 8, AdamW betas of 0.9 and 0.95, and a maximum of 100 epochs. The CCDM with the lowest tuning (12% of development set) MSE from all epochs was selected for testing. Classifier-free guidance was used to increase the adherence of generated synthetic retinal images to conditioning clinicodemographic variables.[5,6] Classifier-Free Guidance is a technique that enables image generation to be controlled by conditioning inputs (such as prompts) without relying on classifier networks. It adjusts the noise-prediction process through a guidance scale, providing versatile control over the degree of adherence to the conditioning inputs.[5,6] Guidance strengths of 2 and 8 were used for the base and super resolution CCDM stages respectively. Other key CCDM sampling parameters were 32 and 64 evaluation steps and sigma churn of 80 and 160 for the base and super-resolution CCDM stages respectively.[7] The chosen hyperparameters, including for guidance strength, evaluation steps, and sigma churn were informed by the hyperparameter sweeps and optimal values reported in the original Imagen and Karras reports.[5,7] Due to computational limitations, conducting our own sweeps was not feasible. Only positive cases were used to augment pDLS development datasets. For the ×2 and ×4 positive-case generation scenarios, the sampled noise vector was varied by changing the seed with overlap with the ×1 cohort, whilst conditioning clinicodemographic variables and other hyperparameters were kept constant.

Prognostic DLS development and hyperparameters

Random augmentations of brightness (± 0.15), contrast (-0.25, +0.25), hue shift (± 7), saturation shift (-63, +127), and horizontal flip were applied during pDLS training for real and synthetic retinal images with a probability of 0.5 and gaussian blur (± 5) with a probability of 0.1.[8] AdamW optimiser was used to minimise the positive class weighted mean binary cross entropy loss with 1:1 weighting between all outcomes. The pDLS batch size was 16, and the base learning rate was 0.0001, with a cosine annealing schedule with a 3 epoch warm start. Other hyperparameters were pre-classification node dropout of 0.2, weight regularisation of 0.0001, and a maximum of 60 epochs. Early stopping criterion of a 5 epoch plateau in the mean tuning (12% of development set) 2-year incident rDR/rM area-under-the receiver operating characteristic (AUROC) was used. The pDLS with the highest mean tuning 2-year incident rDR/rM AUROC was selected for testing. Model training and testing were performed using two Quadro P6000 GPUs and PyTorch v1.9.1.

SUPPLEMENTARY TABLE 1. DIABETIC RETINOPATHY GRADING DEFINITIONS

Grade	Description	Criteria
**Retinopathy**		
**R0**	**No DR**	• No retinal DR lesions
**R1**	**Mild to moderate non-proliferative DR**	• Presence of microaneurysms or retinal haemorrhages<br>• Exudates or cotton wool spots in the presence of DR features
**R2***	**Moderate to severe non-proliferative DR**	• Presence of multiple blot haemorrhages, venous beading or intraretinal microvascular abnormalities
**R3A***	**Proliferative DR**	• New features of proliferative disease, namely, new vessels at the disk or elsewhere, preretinal/vitreous haemorrhage, new preretinal fibrosis or new tractional retinal detachment<br>• Previous treatment for proliferative DR that has not been deemed stable by the treating ophthalmologist<br>• New features indicating reactivation of proliferation, or potentially sight threatening change from fibrous proliferation with respect to previously reference images
**R3S**	**Stable-treated proliferative DR**	• Evidence of peripheral retinal laser treatment and stable retina with respect to reference images taken at or shortly after discharge from the hospital eye service.
**Maculopathy**		
**M0**	**No referable diabetic maculopathy**	• *Referable diabetic maculopathy criteria not met*
**M1***	**Referable diabetic maculopathy**	• Exudate ≤1 disc diameter from the fovea<br>• ≥1/2 a disc area of exudates within the macula<br>• Microaneurysm ≤1 disc diameter from the fovea AND visual acuity ≤6/12

DR=Diabetic retinopathy. *Referable DR or referable maculopathy as per UK national screening committee definitions[1].

SUPPLEMENTARY TABLE 2. DIABETIC EYE SCREENING PROCEDURES

Variable	South-East London Diabetic Eye Screening Programme	Birmingham Diabetic Eye Screening Programme
Country [City]	United Kingdom [London]	United Kingdom [Birmingham]
Setting [Sites]	Diabetic Eye Screening Programme<br>[27 Hospital and Community Sites]	Diabetic Eye Screening Programme<br>[110 Hospital and Community Sites]
Screening Pathway	Routine digital screening	Routine digital screening
Study Duration	Sept 2013 to Dec 2019	Sept 2013 to Dec 2019
Eligibility	12 years and older<br>DM diagnosis<br>Light perception visual acuity or better in at least one eye	12 years and older<br>DM diagnosis<br>Light perception visual acuity or better in at least one eye
Routine Screening Interval	Annual	Annual
Mydriasis	Yes	Yes
Retinal Cameras	Majority: Topcon [NW6, NW8]<br><br>*Minority: Canon CR2, Topcon Triton OCT Fundal Camera*	Majority: Topcon [NW6]<br><br>*Minority: CR-DGi, Nidek AFC-210, Kowa Alpha 8*
Image Type	Colour photographs	Colour photographs
Image Formats	jpg (84%), nef (16%)	jpg (100%)
Imaging Protocol	Two-Field<br>45° fovea-centred (*macula*) & optic-disc-centred (*nasal*)	Two-Field<br>45° fovea-centred (*macula*) & optic-disc-centred (*nasal*)
Native Image Resolutions	[1960 x 1934] to [6000 x 4000]	[1152 x 1728] to [6000 x 4000]
Grading System	1^0 Grader: No DR/DR<br>2^0 Grader: Non-referable/Referable DR, *10% of No DR cases Regraded.*<br>3^0 Grader: Arbitration	1^0 Grader: No DR/DR<br>2^0 Grader: Non-referable/Referable DR, *10% of No DR cases Regraded.*<br>3^0 Grader: Arbitration
Grading Platform Recommendations	Desktop monitor with minimum 1080 pixels vertical resolution	Desktop monitor with minimum 1080 pixels vertical resolution
Quality Assurance & Audit Processes	Local and national audits, key performance indicators, grader qualifications, grader training and testing (~10/year), intergrader agreement reports, 10% no DR regrading to define missed cases, local quality assurance inspections, national guidelines, policies and service specifications.	Local and national audits, key performance indicators, grader qualifications, grader training and testing (~10/year), intergrader agreement reports, 10% no DR regrading to define missed cases, local quality assurance inspections, national guidelines, policies and service specifications.

DM=Diabetes mellitus. DR=Diabetic retinopathy.

SUPPLEMENTARY FIGURE 1. UNCURATED NEGATIVE AND POSITIVE SYNTHETIC RETINAL IMAGE CASE EXAMPLES

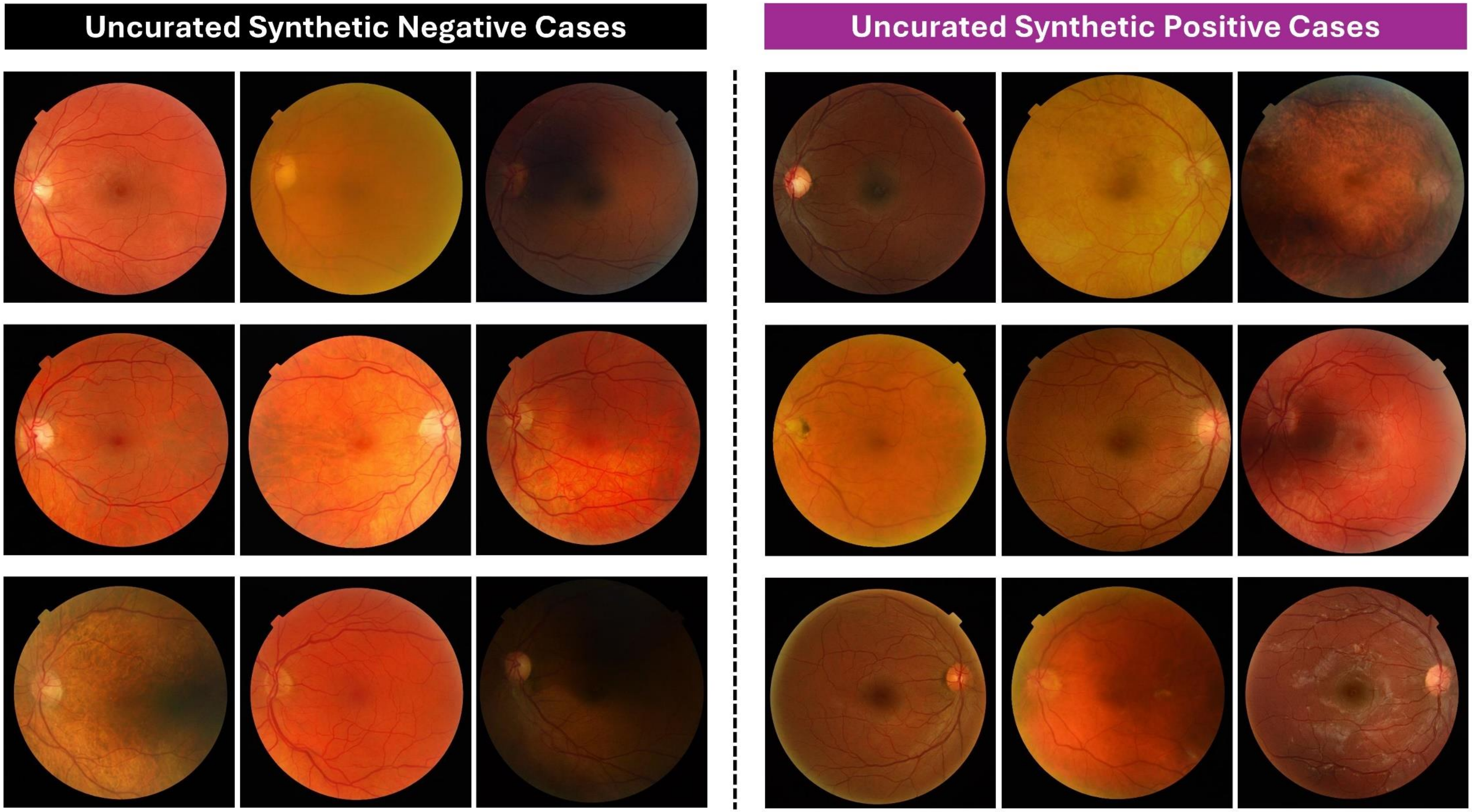

Uncurated synthetic retinal image examples of negative and positive cases. Synthetic retinal images show variations in laterality, clarity, pigmentation and retinal vasculature. There is also variation in the fovea, optic disc morphology, choroidal markings and image quality. Positive cases do not seem to show lesions of mild diabetic retinopathy (DR) such as exudates when this condition variable is set to present. However the vast majority of real retinal images used to train the CCDM had no DR (see Table 1, ~86%). Close inspection of the retinal vascular tree show some areas of anomalous branching or discontinuity.

SUPPLEMENTARY TABLE 3. PROGNOSTIC DLS AUPRC

**Prognostic DLS** [Additional Positive Cases]	**Positive & Negative Training Cases Ratio** [Samples][1]	**2 Year Incident Disease Outcomes**	**SEL-DESP** *(Real Internal Test Set)* *N=9,071 unique eyes / retinal images*			**B-DESP** *(Real External Test Set)* *N=2,842 unique eyes / retinal images*		
			**AUPRC**	**Δ vs pDLS-N**	**% Δ vs pDLS-N**	**AUPRC**	**Δ vs pDLS-N**	**% Δ vs pDLS-N**
pDLS-N [×0]	**1 : 58** [1,351 : 71,208]	rDR/rM	0.142	-	-	0.095	-	-
pDLS-R [×1]	**1 : 26** [2,702 : 71,208]	rDR/rM	0.115	-0.027	-19.0%	0.083	-0.012	-12.6%
pDLS-G [×1]	**1 : 26** [2,702 : 71,208]	rDR/rM	0.131	-0.011	-7.5%	0.131	+0.036	+37.9%
pDLS-G [×2]	**1 : 19** [4,053 : 71,208]	rDR/rM	0.174	+0.032	+22.5%	0.151	+0.056	+58.9%
pDLS-G [×4]	**1 : 11** [6,755 : 71,208]	rDR/rM	0.158	+0.016	+11.3%	0.124	+0.029	+30.5%

[1]Ratios and number of positive and negative cases (eyes) used during pDLS training. AUPRC=Area-Under-the Precision Recall Curve, 95%CI=95% Confidence Interval, SEL-DESP=Southeast London diabetic eye screening programme, B-DESP=Birmingham diabetic eye screening programme. Δ=Difference. DR=Diabetic Retinopathy, rDR/rM=Referable diabetic retinopathy or maculopathy (2-year incident), pDLS-N=Native unaugmented image prognostic DLS, pDLS-R=Resampled positive case augmented prognostic DLS, pDLS-G=Generated positive case augmented prognostic DLS *Significant if $p<0.05$. Underlined=Numerical improvement compared to pDLS-N.

SUPPLEMENTARY REFERENCES

1. PHE. NHS Diabetic Eye Screening Programme: Grading definitions for referable disease. 2021. https://www.gov.uk/government/publications/diabetic-eye-screening-retinal-image-grading-criteria/nhs-diabetic-eye-screening-programme-grading-definitions-for-referable-disease (accessed 13/10/2021.

2. PHE. NHS public health functions agreement 2019-20: Service specification no.22 (NHS Diabetic Eye Screening Programme). 2019. https://www.england.nhs.uk/wp-content/uploads/2017/04/Service-Specification-No.22-NHS_Diabetic_eye_screening.pdf.

3. PHE. The management of grading quality: Good practice in the quality assurance of grading. 2016. https://assets.publishing.service.gov.uk/media/5a80521b40f0b62305b8a76c/The_Management_of_Grading.pdf.

4. PHE. NHS Diabetic Eye Screening Programme: Overview of patient pathway, grading pathway, surveillance pathways and referral pathways. 2017. https://www.gov.uk/government/publications/diabetic-eye-screening-pathways-patient-grading-referral-surveillance (accessed 07/06/2022.

5. Saharia C, Chan W, Saxena S, et al. Photorealistic Text-to-Image Diffusion Models with Deep Language Understanding. 2022: arXiv:2205.11487.

6. Ho J, Salimans T. Classifier-Free Diffusion Guidance2022. https://ui.adsabs.harvard.edu/abs/2022arXiv220712598H (accessed July 01, 2022).

7. Karras T, Aittala M, Aila T, Laine S. Elucidating the Design Space of Diffusion-Based Generative Models2022. https://ui.adsabs.harvard.edu/abs/2022arXiv220600364K (accessed June 01, 2022).

8. Krause J, Gulshan V, Rahimy E, et al. Grader Variability and the Importance of Reference Standards for Evaluating Machine Learning Models for Diabetic Retinopathy. *Ophthalmology* 2018; **125**(8): 1264-72.
